# A novel tumor doubling time-related immune gene signature for prognosis prediction in hepatocellular carcinoma

**DOI:** 10.1186/s12935-021-02227-w

**Published:** 2021-10-09

**Authors:** Genhao Zhang, Lisa Su, Xianping Lv, Qiankun Yang

**Affiliations:** 1 Department of Blood Transfusion, The First Affiliated Hospital of Zhengzhou University, Zhengzhou, China; 2 Department of Genetic and Prenatal Diagnosis Center, The First Affiliated Hospital of Zhengzhou University, Zhengzhou, China

**Keywords:** HCC, Prognosis, Immune, Tumor doubling time, CLEC1B

## Abstract

**Background:**

Hepatocellular carcinoma (HCC) has become a global health issue of wide concern due to its high prevalence and poor therapeutic efficacy. Both tumor doubling time (TDT) and immune status are closely related to the prognosis of HCC patients. However, the association between TDT-related genes (TDTRGs) and immune-related genes (IRGs) and the value of their combination in predicting the prognosis of HCC patients remains unclear. The current study aimed to discover reliable biomarkers for anticipating the future prognosis of HCC patients based on the relationship between TDTRGs and IRGs.

**Methods:**

Tumor doubling time-related genes (TDTRGs) were acquired from GSE54236 by using Pearson correlation test and immune-related genes (IRGs) were available from ImmPort. Prognostic TDTRGs and IRGs in TCGA-LIHC dataset were determined to create a prognostic model by the LASSO-Cox regression and stepwise Cox regression analysis. International Cancer Genome Consortium (ICGC) and another cohort of individual clinical samples acted as external validations. Additionally, significant impacts of the signature on HCC immune microenvironment and reaction to immune checkpoint inhibitors were observed.

**Results:**

Among the 68 overlapping genes identified as TDTRG and IRG, a total of 29 genes had significant prognostic relevance and were further selected by performing a LASSO-Cox regression model based on the minimum value of λ. Subsequently, a prognostic three-gene signature including HECT domain and ankyrin repeat containing E3 ubiquitin protein ligase 1 (HACE1), C-type lectin domain family 1 member B (CLEC1B), and Collectin sub-family member 12 (COLEC12) was finally identified by stepwise Cox proportional modeling. The signature exhibited superior accuracy in forecasting the survival outcomes of HCC patients in TCGA, ICGC and the independent clinical cohorts. Patients in high-risk subgroup had significantly increased levels of immune checkpoint molecules including PD-L1, CD276, CTLA4, CXCR4, IL1A, PD-L2, TGFB1, OX40 and CD137, and are therefore more sensitive to immune checkpoint inhibitors (ICIs) treatment. Finally, we first found that overexpression of CLEC1B inhibited the proliferation and migration ability of HuH7 cells.

**Conclusions:**

In summary, the prognostic signature based on TDTRGs and IRGs could effectively help clinicians classify HCC patients for prognosis prediction and individualized immunotherapies.

**Supplementary Information:**

The online version contains supplementary material available at 10.1186/s12935-021-02227-w.

## Background

Hepatocellular carcinoma (HCC) is considered an aggressive malignancy, and has become a global health issue of wide concern due to its high prevalence and poor therapeutic efficacy [1]. The incidence and mortality of HCC have emerged rapidly on the background of increased alcohol abuse, cirrhosis, aflatoxin exposure, diabetes, metabolic syndrome, and obesity. With the rapid developments in immunotherapy, HCC patients could access potential treatment strategies including Sorafenib, Lenvatinib, and Atezolizumab [2], while not all the patients could benefit from these immune-based therapies due to the complex immune status of HCC [3–5]. Tumor doubling time (TDT), a classic biomarker reflecting tumor growth patterns, has some implications for general management of tumor patients including anticancer treatment reactivity and outcome, tumor histologic type prediction, and best surveillance intervals determination [6–9]. When gradual and predictable tumor growth was determined based on TDT, patients with indolent or aggressive tumors could be identified at an early stage and receive follow-up treatment to improve final survival, which may reduce the mental and financial burden caused by overdiagnosis or missed diagnosis [9–11]. What's more, TDT was highly associated with tumor vascular invasion and recurrence [12] and could be regarded as a reliable predictor of sorafenib's therapeutic effect [7], suggesting that TDT might have a significant relationship with the cancer immune status. Furthermore, immunotherapy is becoming the new standard of treatment for advanced stages, considering its increasing use worldwide to treat HCC patients, although the inadequate immune response has been a growing concern [13]. However, the association between TDT-related genes (TDTRGs) and immune-related genes (IRGs) and the value of their combination in predicting the prognosis of HCC patients remains unclear. In the present study, a novel prognostic signature based on currently available TDTRGs and IRGs could efficiently classify HCC patients for prognosis and individualized immunotherapies.

## Materials and methods

### Public datasets and generation of TDTRGs and IRGs

The mRNA expression data and clinical characteristics of HCC patients from three publicly available datasets including TCGA-LIHC, GSE54236, and ICGC (LIRI-JP) were incorporated into the present study. Genes with a cut-off criterion of adjust *P* value < 0.01 and Pearson correlation coefficient > 0.3 in GSE54236 were considered TDTRGs. IRGs were available from the Immunology Databases and Analysis Portal (ImmPort) database (https://www.immport.org/home) [14].

### Prognostic risk score model construction and functional analysis

The univariable Cox regressions were first performed to calculate the association between overlapped genes of TDTRGs and IRGs and survival outcomes in TCGA-LIHC cohort. Then LASSO-Cox regression method and stepwise Cox regression analysis were performed to evaluate the above prognosis-related genes and establish a prognostic signature. A risk score was finally established based on the basis of linearly combining the formula below with the mRNA expression level multiplied by the multivariate Cox regression coefficient (β) model. Risk score = (β_mRNA1_ × mRNA1) + (β_mRNA2_ × mRNA2) + … + (β_mRNAn_ × mRNAn). We stratified patients in TCGA dataset into two subgroups due to the optimal risk score threshold. The predictive power and independence of the prognostic signature in TCGA were assessed by ROC analysis, Kaplan–Meier survival analysis, and Cox proportional hazards regression analysis. Gene set enrichment analysis (GSEA) between the two subgroups was performed to identify the significantly alerted Hallmarks with FDR < 0.05. Annotated gene set h.all.v7.2.symbol.gmt (Hallmarks) was chosen as the reference gene set.

### Clinical specimens and quantitative real-time PCR (qRT-PCR) analysis

Fresh frozen tumor tissues from previously collected HCC patients were selected as an independent validation cohort [15]. qRT-PCR was used to detect the mRNA levels of genes in the model [16]. After the relative mRNAs expression levels were normalized to *β*-ACTIN and log_2_ transformed, patients were stratified into two subgroups according to the above formula. Primer sequences are showed in Additional file 3: Table S1.

### Immune status calculation and immune infiltrates analysis

The immune status of each sample was assessed by applying the ESTIMATE algorithm [17] to the TCGA cohort and calculating immune and stromal scores, and the association between risk scores and immune, stromal scores were analyzed by Pearson correlation analysis. To explore the impacts of the prognostic model on immunotherapies, we calculated the relationship among risk score and 15 potentially available targeted immune checkpoint genes in TCGA-LIHC, including CCL2, CD274, CD276, CD4, CTLA4, CXCR4, IL1A, IL6, LAG3, PDL1, PDL2, TGFB1, OX40, CD137 and CDX40L [18]. Furthermore, to assess the potential association between prognostic signature and tumor-infiltrating immune cells (TIICs) in the HCC microenvironment, the TCGA database was used to measure the abundance ratios of 22 types of TIICs through CIBERSORT [19] (http://cibersort.stanford.edu/). Finally, the predictive ability of significantly changed TIICs was assessed by Kaplan–Meier survival analysis.

### Genetic alterations and TMB analysis

The mutation and CNA data of 361 HCC patients were downloaded from TCGA to analyze the difference of genetic alterations between the high- and low-risk score subgroups with R package “maftools”, and the tumor mutation burden (TMB) of each patient was subsequently assessed.

### Cell culture, plasmids construction, and cell infection

HuH7 cells purchased from ATCC were cultured in recommended DMEM medium (Sangon Biotech, China) with 10% fetal bovine serum (FBS, Sangon Biotech, Shanghai, China) in 100% humidity at 37 °C with 5% CO_2_. Lentiviral vector encoding the full-length human CLEC1B DNA sequence (Ubi- CLEC1B -3FLAG-SV40-EGFP-IRES-puromycin) and empty vector were selected for generating stable overexpressing (OE) and negative control (OE-NC) stable cell lines using lipofectamine™ 3000 transfection reagent (Invitrogen, Carlsbad, USA) according to the manufacturer’s instructions.

### Cell proliferation and migration assay

The viability of cells was assessed through the cell counting kit-8 (CCK-8, Sangon Biotech, Shanghai, China) in accordance with the manufacturer’s instructions. For the CCK-8 assay, in short, cells were seeded in 96-well cell culture clusters at a density of 1 × 10^5^ cells per well and cultured for 1 day, 2 days, and 3 days, respectively. After culturing, 10 μL CCK-8 solution was added into each well, and then the absorbance was detected at a wavelength of 450 nm within 4 h with a microplate reader. For the wound-healing assay, cells were seeded in 6-well plates and cultured to approximately 80% confluence in serum-free medium and then cell monolayers were scratched with a sterile pipette tip. Cells were cultured in DMEM medium with 10% FBS for the next 24 h after removal of cell debris by PBS washing. The area of the wound width was measured after photographing the wound width of the cell monolayers. Three independent duplicates needed to ensure the accuracy in this assay and the wound closure rate was calculated as [1—(wound area / original wound area)] from photographs. For the transwell assay, after transwell filters were coated with Matrigel, cultured cells were resuspended in 200 μL serum-free DMEM at a density of 1 × 104 cells per mL, and plated into the transwell inserts while the wells were filled with 500 μL DMEM supplemented with 10% FBS. After incubated at 37℃ for 48 h, cells attached to the downside of the transwell filters were stained with 0.1% crystal violet in PBS for 15 min and counted under microscopy at 200 × magnification.

### Statistical analysis

Categorical data were compared with Pearson chi-square test or Fisher exact test whenever appropriate, and quantitative variables were analyzed using independent-samples *t* test. ROC curve analysis and Kaplan–Meier survival analysis were performed to assess the prediction performance of survival outcomes with R software (Version 4.0.3). Cox proportional model was performed to analyze the relationship between prognostic signature and survival outcomes, together with other clinical features. Clinical characteristics of HCC patients in TCGA, ICGC and clinical validation cohorts were showed in Additional file 3: Table S2. Results were considered statistically significant when *P* value < 0.05.

## Results

### Identification of overlapped genes in TDTRGs and IRGs

**Fig. 1:**
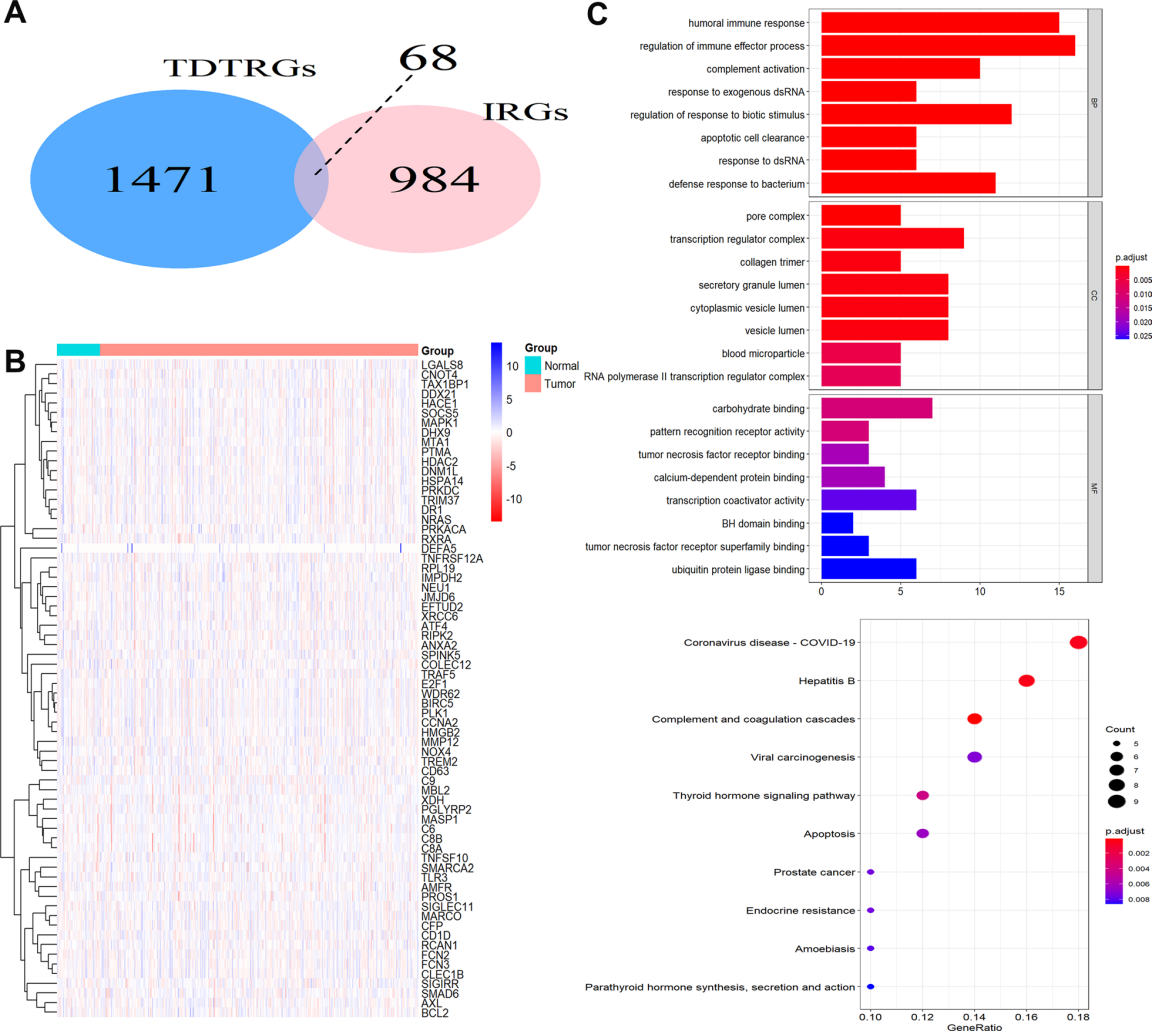
Identification of overlapped genes in TDTRGs and IRGs. **A** 68 overlapped genes in TDTRGs and IRGs. **B** The expression of overlapped genes in TCGA-LIHC. **C** GO and KEGG analysis of overlapped genes

With the cut-off criterion of adjust *P* value < 0.01 and Pearson correlation coefficient > 0.3, 1539 genes in GSE54236 were considered TDTRGs. Then 68 genes were identified as overlapped genes of TDTRGs and IRGs (Fig. 1A). The expression levels of the 68 overlapping genes in normal and tumor tissues in the TCGA-LIHC dataset were shown in Fig. 1B. Results of Go and KEGG analysis indicated that these overlapped genes were majorly associated with immune response and inflammation (Fig. 1C).

### Establishment of a prognosis-related signature in TCGA

**Fig. 2:**
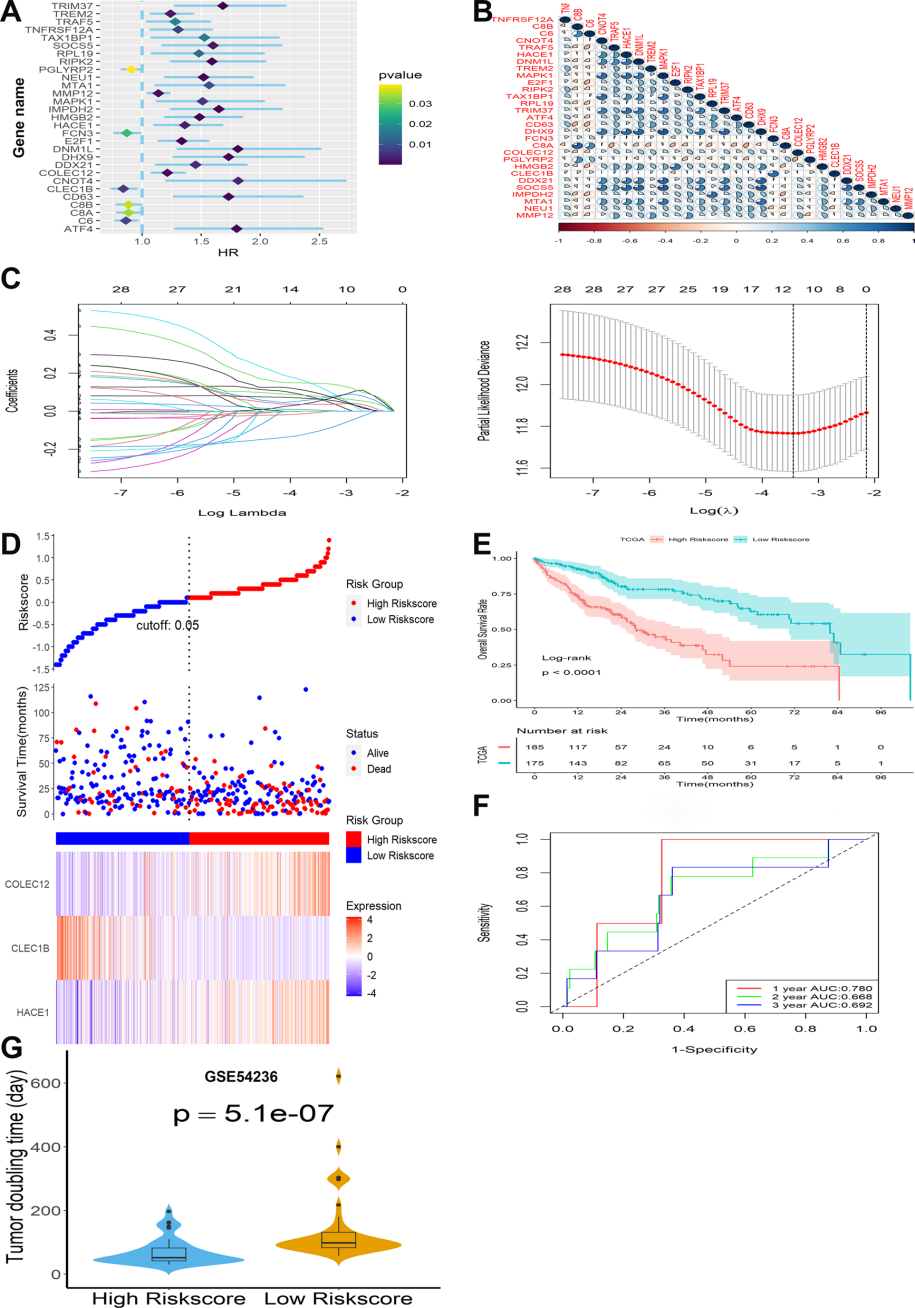
Construction and survival analysis of three-gene signature in TCGA. **A** Forest plots showing the results of 29 prognosis-related overlapping genes to univariate Cox analysis. **B** Correlation analysis of 29 overlapping genes. **C** Adjustment of parameter selection in LASSO-Cox analysis by 10 cross-validations. **D** Distribution of risk scores, OS status, and gene expression profiles. **E** Kaplan–Meier survival plot. **F** Characteristics in ROC analysis for predicting 1, 2, and 3-year OS rates. **G** TDT difference between the two subgroups

As calculated by univariable Cox regression, 29 of the 68 overlapped genes had significant prognostic relevance (Fig. 2A) and were further analyzed. The correlations between these prognostic genes were shown in Fig. 2B. Then the most valuable prognostic genes among the 29 genes above were selected by performing the LASSO-Cox regression model based on the minimum value of λ (Fig. 2C) and a prognostic three-gene signature was finally identified via a stepwise Cox proportional model. Risk score = (0.2984276 × HACE1) + (0.1782599 × COLEC12) – (0.2154380 × CLEC1B). Risk scores for HCC patients were calculated with the above formula, and patients were stratified into high- or low-risk subgroups with an optimal risk score threshold (Fig. 2D). The association between risk score and clinical characteristics including age, gender, grade, stage, vascular invasion, value of AFP, cirrhosis, and tumor status were evaluated. The results revealed that higher risk scores were linked to advanced TNM stage, later grade, later T stage, and recurrence (Additional file 1: Figure S1). Kaplan–Meier survival analysis revealed that patients with higher risk scores were significantly relevant to poorer survival outcomes (Fig. 2E). In addition, further stratified survival analysis was applied for different clinical characteristics, and the results demonstrated that this prognostic model could further differentiate patients with different clinical characteristics including age, vascular invasion, grade, recurrence, TNM stage, gender, and AFP value (Additional file 2: Figure S2). Finally, ROC analysis revealed that this signature had a good prognostic performance with AUCs at 1-, 2-, 3-year of 0.780, 0.668, 0.692 (Fig. 2F). Finally, the relationship between risk score and TDT was analyzed, and we found that HCC patients in the high-risk group had significantly shorter TDT (Fig. 2G).

### Verification of the signature in ICGC cohort and clinical cohort

**Fig. 3:**
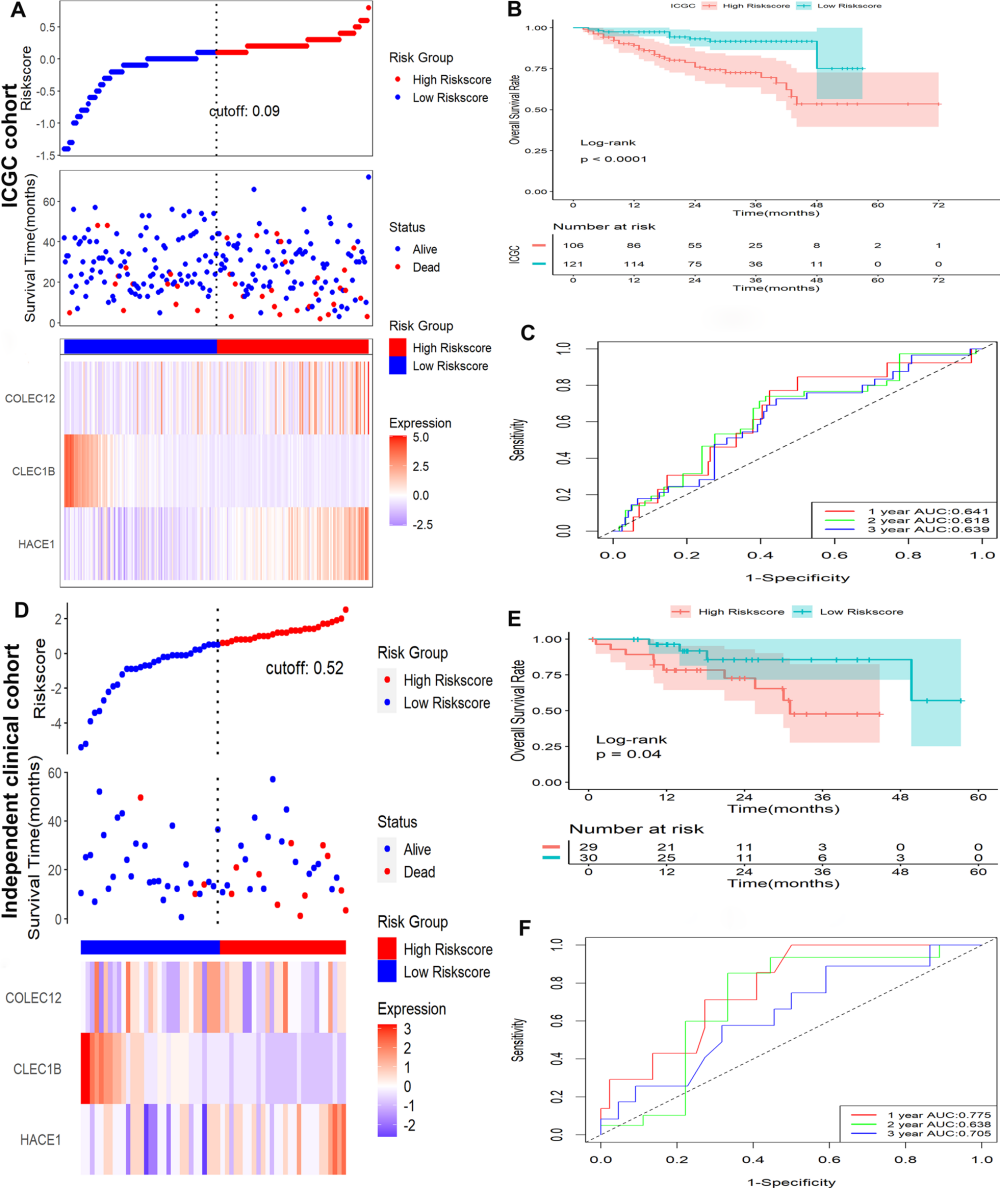
Verification of this signature in ICGC and clinical cohort. **A** Distribution of risk scores, OS status, and gene expression profiles in ICGC. **B** Kaplan–Meier survival plot in ICGC. **C** Characteristics in ROC analysis for predicting 1, 2, and 3-year OS rates in ICGC. **D** Distribution of risk scores, OS status, and gene expression profiles clinical cohort. **E** Kaplan–Meier survival plot clinical cohort. **F** Characteristics in ROC analysis for predicting 1, 2, and 3-year OS rates clinical cohort

To validate the signature, the ICGC dataset and a clinical cohort were applied as validation cohorts. Risk scores of patients were calculated with the same formula, and patients were stratified into high- or low-risk subgroups in the ICGC cohort (Fig. 3A) and the clinical cohort (Fig. 3D). Kaplan–Meier survival analysis revealed that patients with higher risk scores were prominently relevant to poorer OS rates in the ICGC cohort (Fig. 3B), as well as in the clinical cohort (Fig. 3E). ROC analysis revealed that this signature had a good prognostic performance with AUCs at 1-, 2-, 3-year of 0.641, 0.618, 0.639 in the ICGC cohort (Fig. 3C) and 0.775, 0.638, 0.705 in the clinical cohort (Fig. 3F), respectively.

### Establishment of a nomogram model in TCGA

**Fig. 4:**
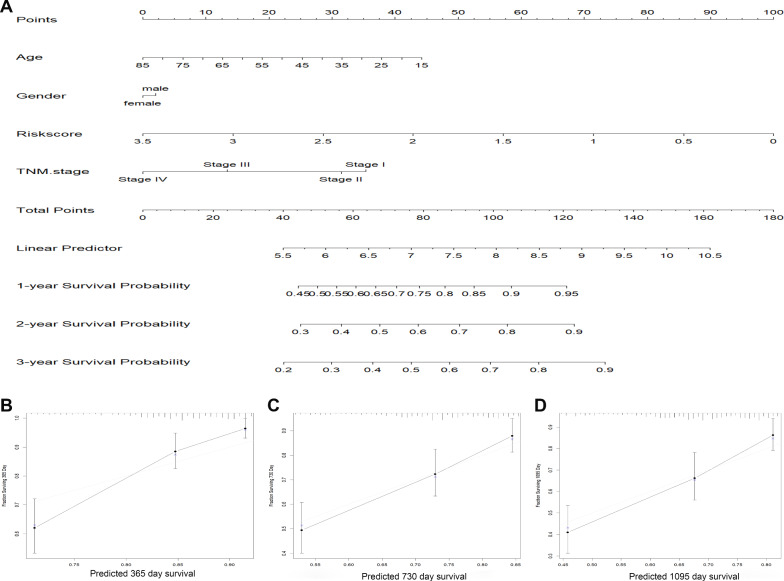
Predictive significance of signature verified in nomogram model. **A** A nomogram combining the three-gene signature. **B**–**D** The calibration plots for 1, 2, 3 years survival probabilities

To investigate the coefficient prediction efficiency of this signature, a nomogram model was established in the TCGA dataset, and the result revealed that the nomogram with a C-index of 0.713 could help us provide a quantitative method for predicting the 1-, 2-, 3-year survival rate accurately (Fig. 4A). The overlap between the forecasted and actual probabilities of 1-, 2-, 3-year survival rate in the calibration curves indicated good agreement (Fig. 4B–D).

### Functional analysis and immune status calculation

**Fig. 5:**
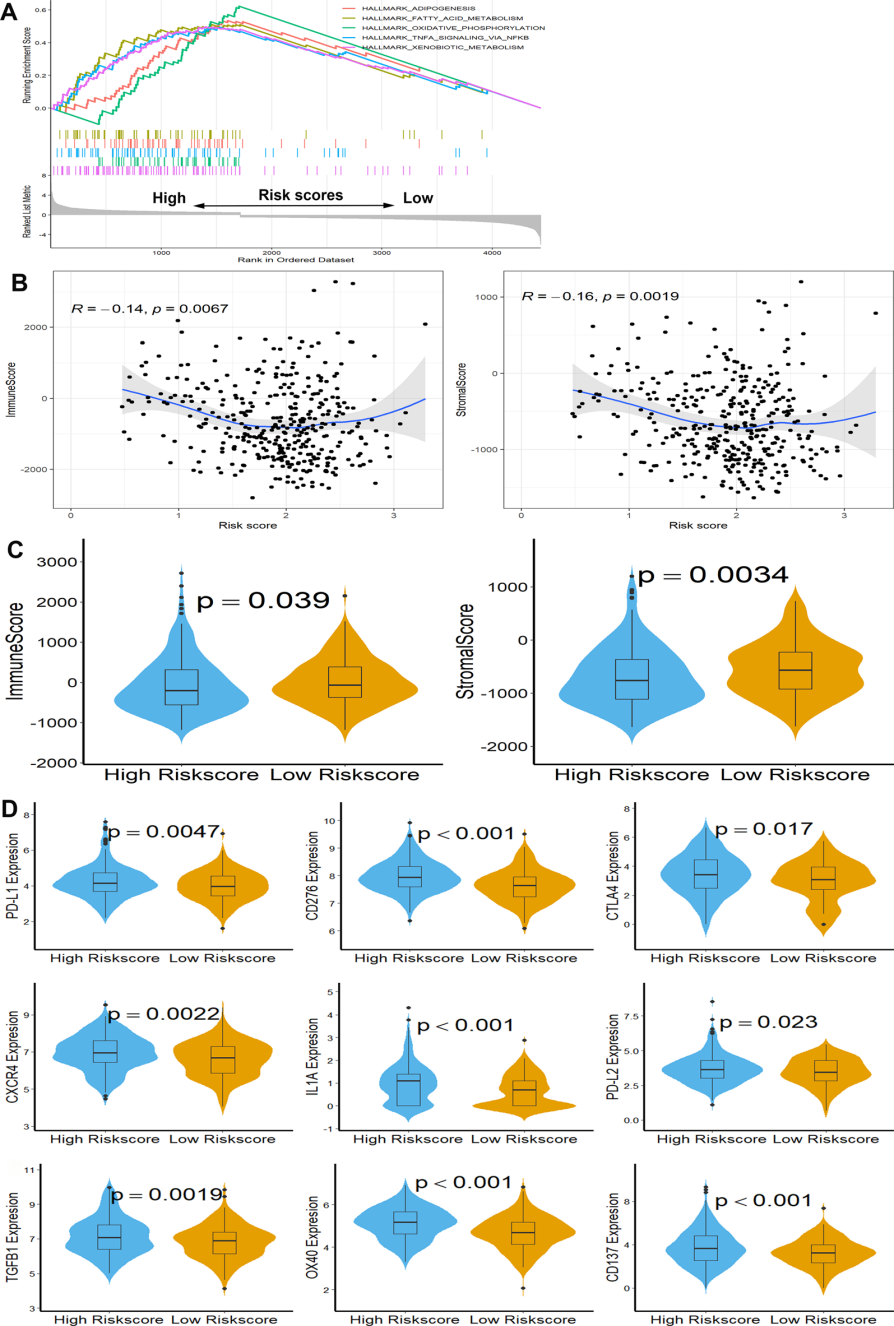
Functional analysis, immune status calculation, and levels of immune checkpoint genes in two subgroups. **A** GSEA is based on the three-gene signature. **B** Correlation analysis of risk scores, immune scores, and stromal scores. **C** Difference analysis of immune scores and stromal scores in two subgroups. **D** Expression levels of immune checkpoint genes in two subgroups

To investigate the essential molecular mechanisms within this three-gene signature, Gene Set Enrichment Analysis (GSEA) was performed to identify significantly alerted biological processes and pathways. Annotated gene set h.all.v7.2.symbol.gmt (Hallmarks) was chosen as the reference gene set. Five Hallmarks with FDR < 0.05 were enriched in this signature, including adipogenesis, fatty acid metabolism, oxidative phosphorylation, TNFA signaling via NFKB, and xenobiotic metabolism (Fig. 5A). According to the results of the ESTIMATE algorithm, risk scores was significantly associated with immune scores, as well as stromal scores (Fig. 5B), and patients in the low-risk subgroup had higher immune scores and stromal scores when compared with patients in the high-risk subgroup (Fig. 5C), indicating that this signature was closely related to tumor immune status. In the following, the expression levels of 15 potentially targetable immune checkpoint genes were compared between the two subgroups in the TCGA database, and results showed that patients in the high-risk subgroup had significantly increased PD-L1, CD276, CTLA4, CXCR4, IL1A, PD-L2, TGFB1, OX40 and CD137 (Fig. 5D), indicating that immune checkpoint inhibitors (ICIs) treatment were more effective for patients in high-risk subgroup.

### Immune infiltrates analysis

**Fig. 6:**
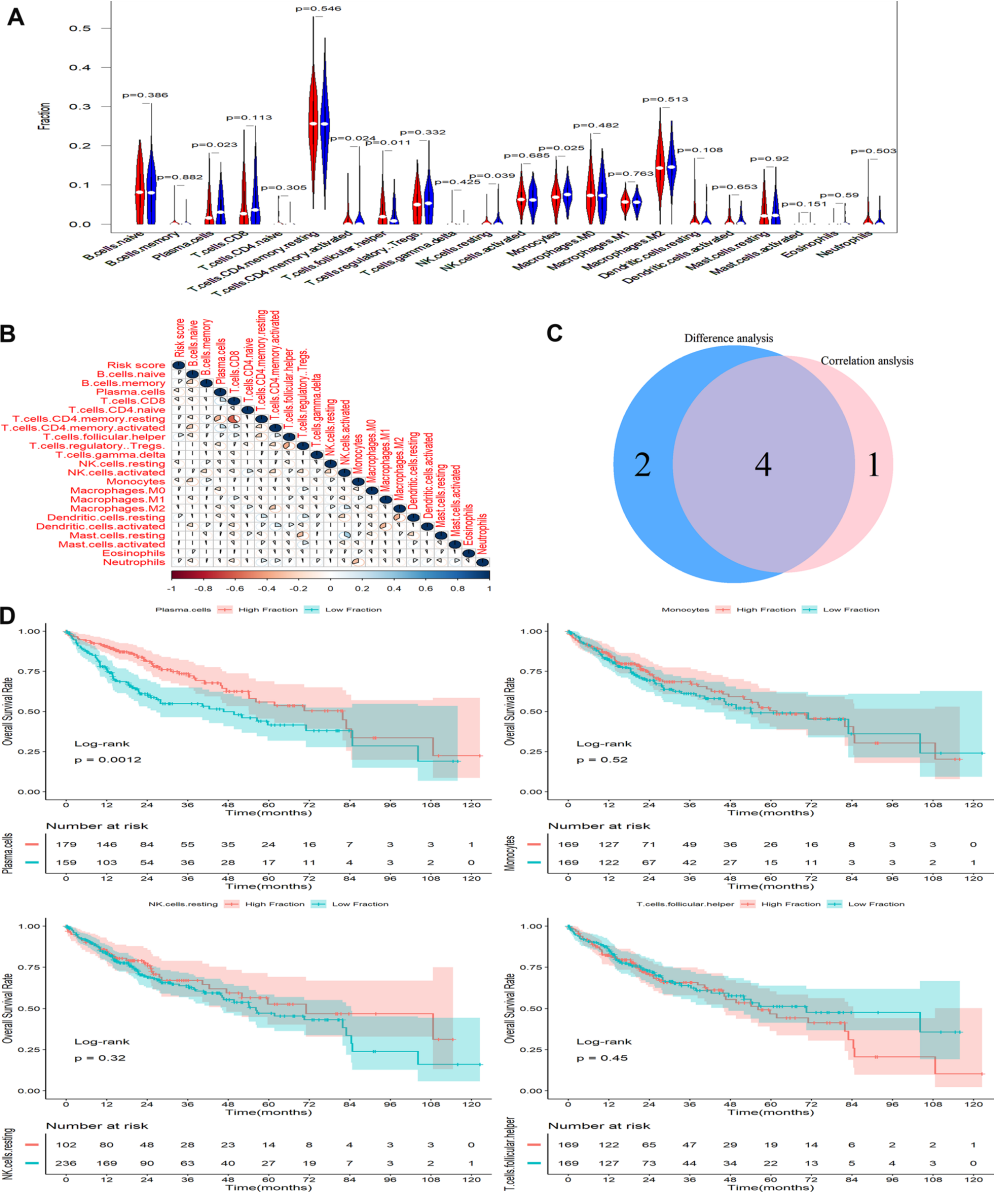
Immune infiltrates analysis. **A** Violin plot showing the abundance differentiation of 22 TIICs in two subgroups. **B** Correlation analysis of risk scores and abundance of 22TIICs. **C** Venn plot showing four TICs that were differentially expressed and correlated with risk scores. **D** Kaplan–Meier survival plot for the four TIICs

Based on the CIBERSORT algorithm, the differences and correlations of 22 types of TIICs in two subgroups in TCGA were assessed by Wilcoxon signed-rank test and Pearson correlation analysis, respectively. Difference analysis demonstrated that HCC patients in the low-risk score subgroup had modestly increased ratios of plasma cells, CD4 memory resting T cells, resting NK cells, and monocytes, while patients in high-risk score subgroup had significantly elevated ratios of follicular helper T cells (Fig. 6A). Pearson correlation analysis indicated that six types of TIICs were significantly associated with the risk scores, including plasma cells, CD 8 T cells, resting NK cells, follicular helper T cells, resting dendritic cells, and monocytes (Fig. 6B). Furthermore, plasma cells, resting NK cells, follicular helper T cells, and monocytes were considered as overlapping TIICs (Fig. 6C). Among the four overlapped TIICs, unfortunately, Kaplan–Meier survival analysis revealed that only plasma cells were prominently relevant to poor survival outcomes in HCC patients (Fig. 6D).

### Genetic alterations and TMB analysis

**Fig. 7:**
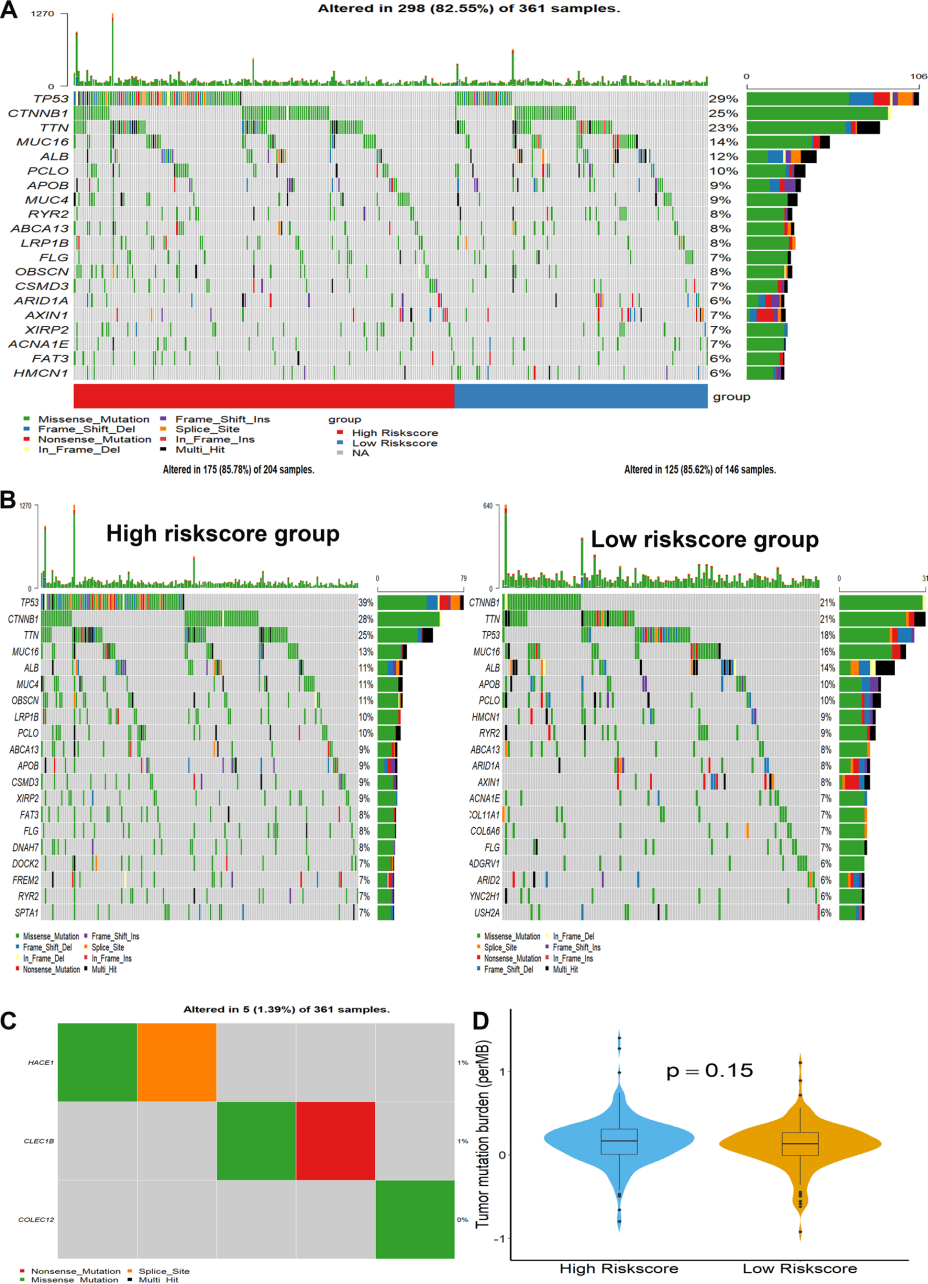
Somatic mutation and TMB analysis. **A** Mutation profile of HCC patients in TCGA cohort. **B** Oncoplots of mutated genes in two subgroups. **C** Genetic alterations of the three genes. **D** Difference analysis of TMB in two subgroups

The results of genetic alterations analysis indicated that the top 10 most significantly mutated genes were TP53, TTN, CTNNB1, MUC16, ALB, PCLO, APOB, MUC4, RYR2, and ABCA13 in the TCGA cohort (Fig. 7A). In addition, the mutation rates of the above genes were remarkably different in the two subgroups (Fig. 7B). Both HACE1 and CLEC1B had a mutation in 1% of HCC samples, while less than 1% mutation rate was found for COLEC12 in HCC patients (Fig. 7C). Subsequently, the TMB of each patient was assessed. However, no significant difference in TMB was found between the two subgroups (Fig. 7D).

### Expression levels of genes in model

**Fig. 8:**
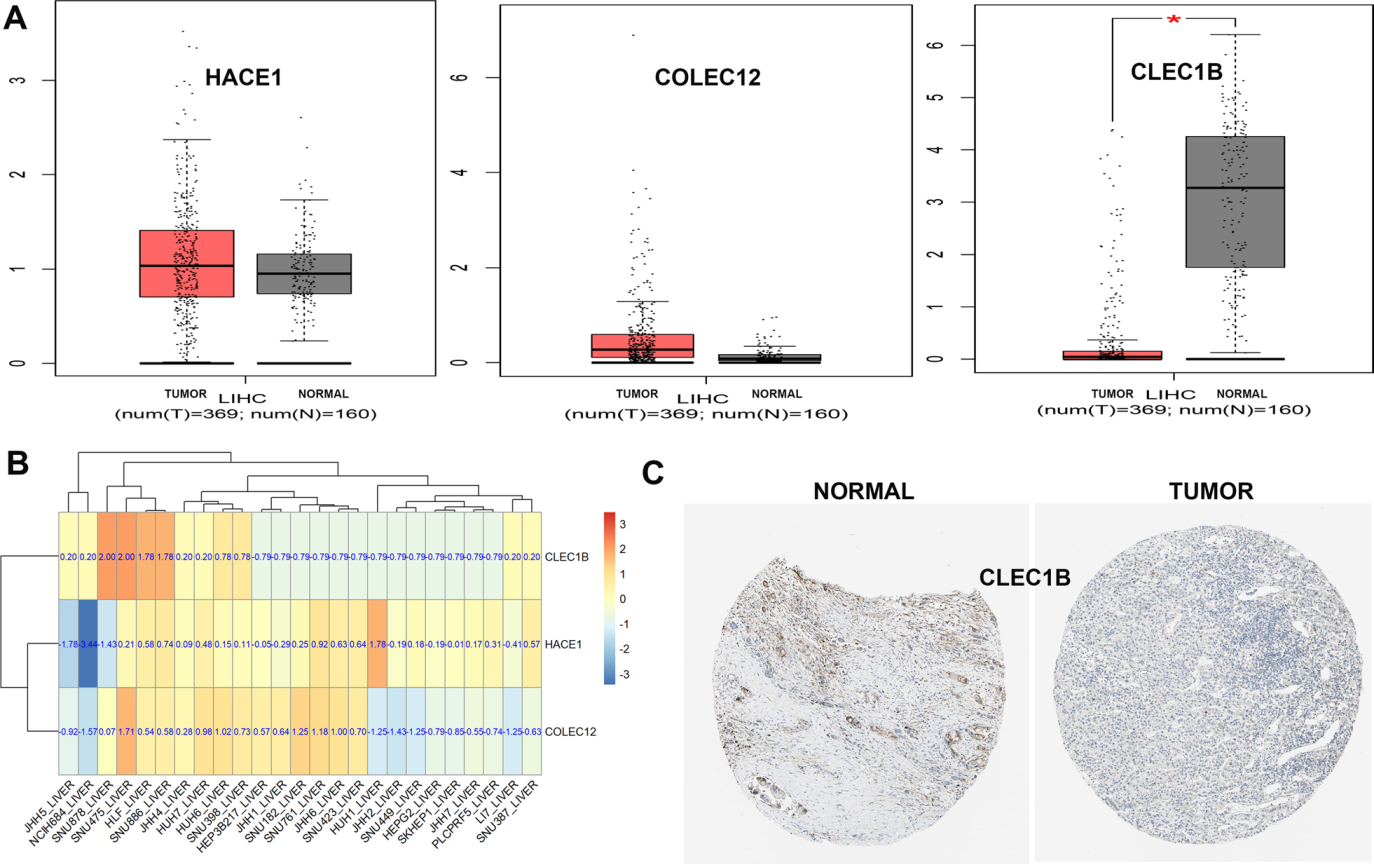
Expression levels of the three genes. The mRNA expression levels of the three genes in GEPIA (**A**) and CCLE (**B**). *p < 0.05. **C** The protein expression levels of CLEC1B in HPA

Differential mRNA expression of the three genes between tumor and normal tissues was explored in Gene Expression Profiling Interactive Analysis (GEPIA) [20] (http://gepia.cancer-pku.cn/) and we found that only CLEC1B differed significantly and therefore was focused on further analysis (Fig. 8A). Differences in mRNA expression of the three genes in HCC cell lines were explored in Cancer Cell Line Encyclopedia (CCLE) [21] (https://portals.broadinstitute.org/ccle) and all of these genes were significantly different across HCC cell lines (Fig. 8B). Finally, the protein expression levels of CLEC1B between tumor and normal tissues were explored in Human Protein Atlas (HPA, www.proteinatlas.org), and a significant difference was detected (Fig. 8C).

### Inhibition of overexpressed CLEC1B on cell proliferation and migration ability

**Fig. 9:**
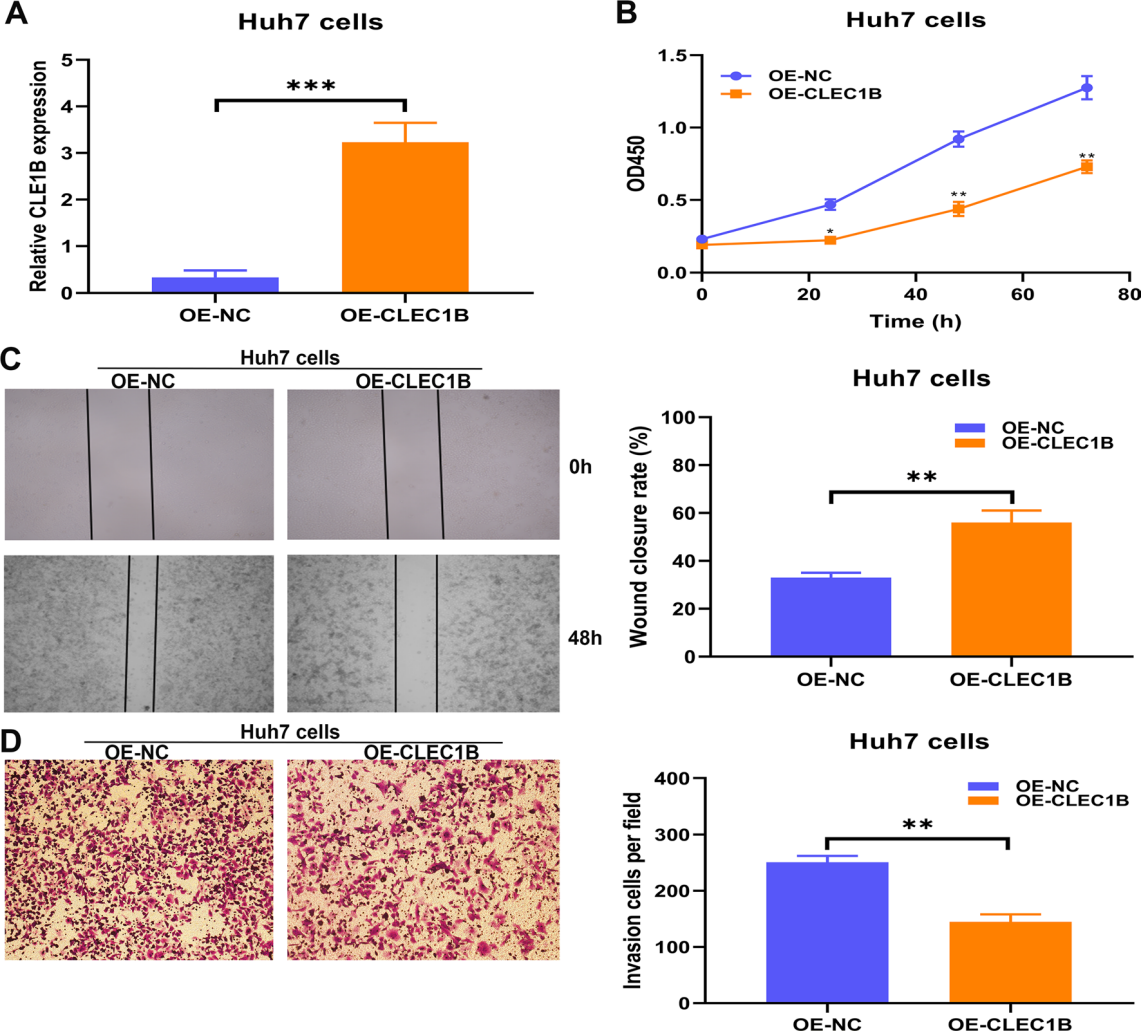
Overexpressed CLEC1B inhibited cell proliferation and migration in Huh7 cells. **A** Lentivirus vector encoding the full-length human CLEC1B DNA sequence was used to manipulate CLEC1B expression. **B** CCK-8 assay, **C** Wound-healing assay, and **D** transwell assay were used to detect the effect of overexpressed CLEC1B on cell proliferation and migration ability. *p < 0.05, **p < 0.01, ***p < 0.001

After generation of stable overexpression (OE) and negative control (OE-NC) cell lines, CLEC1B expression in HuH7 cells was confirmed by qRT-PCR (Fig. 9A). To investigate the influence of CLEC1B on the malignant growth and migration potential, cell proliferation assay, wound-healing assay, and transwell assay were performed. For cell proliferation ability, the results of CCK-8 showed that the proliferation rate of up-regulated CLEC1B was significantly lower than controls in HuH7 cells (Fig. 9B), indicating that CLEC1B inhibited the proliferation ability of HuH7 cells. For migration ability, wound-healing assay showed that CLEC1B overexpression remarkably inhibited migration ability in HuH7 cells (Fig. 9C), as did the result of transwell assay (Fig. 9D). All the results above indicated that CLEC1B might inhibit the proliferative and migration potential of HCC cells.

## Discussion

Tumor doubling time could effectively reflect the growth pattern of tumors, which was crucial for arranging times for tumor detection and chemotherapy interventions [7] and was valuable for predicting the trend of tumor metastasis [22]. Previous studies showed that the tumor growth patterns differed significantly between indolent and invasive tumors [6, 23], indicating that we can initially determine the indolent or aggressive biology based on TDT. Besides, even in the same tumor, such as HCC, an aggressive malignancy with a very short five-year survival time, the tumor growth patterns varied significantly due to the HBV or HCV virus infection, non-viral liver disease, and individual variability. On the other hand, immunotherapy for HCC patients within the tumor immune microenvironment (TIME) has been greatly developed in recent years. Some advanced HCC patients could benefit from these immune checkpoint inhibitors therapies and exhibit favorable survival outcomes [24–27]. Therefore, it is necessary to study prediction models based on TDT and TIME for more accurate HCC monitoring and treatment in clinical practice.

In the present study, tumor doubling time-related genes (TDTRGs) were acquired from GSE54236 by using the Pearson correlation test and immune-related genes (IRGs) were available from ImmPort. Prognostic TDTRGs and IRGs obtained from univariable Cox regression analysis in TCGA-LIHC dataset were determined to create a prognostic model by the LASSO-Cox regression and stepwise Cox regression analysis. Three genes (HACE1, CLEC1B, and COLEC12) were involved in the model. International Cancer Genome Consortium (ICGC) and another cohort of individual clinical samples acted as external validations demonstrated that the signature exhibited superior accuracy in forecasting the survival outcomes and TDT in HCC patients. More importantly, this three-gene signature was an independent risk factor for HCC patients when other clinical factors in the three cohorts were considered. Additionally, significant impacts of the signature on the HCC immune microenvironment and reaction to immune checkpoint inhibitors were observed. Finally, we found overexpressed CLEC1B could inhibit the proliferation and migration of Huh7 cells, which is consistent with the prevision research [28]. In conclusion, the prognostic signature based on TDTRGs and IRGs could efficiently classify HCC patients for prognosis prediction and individualized immunotherapies, and CLEC1B might be an immunotherapy target in the future.

HECT domain and ankyrin repeat containing E3 ubiquitin protein ligase 1 (HACE1) is an E3 ubiquitin ligase located on chromosome 6q21 that plays a crucial role in tumor biology and is closely associated with survival outcomes. HACE1 could act as a tumor suppressor in various human malignancies, including lung cancer [29], HCC [30], breast cancer [31], osteosarcoma [32], colorectal cancer [33], gastric cancer [34], Leukemia [35] and Wilms tumors [36]. For example, in lung cancer, HACE1 deletion could promote KRas^G12D^-driven lung cancer progression by modulating the tumorigenic activation of RAC-family GTPases [29]; HACE1 could accelerate autophagic flux to inhibit tumor growth by ubiquitinating the autophagy receptor and could serve as an autophagy-related target for immunotherapeutic intervention [37]; HACE1 reduced the accumulation of HIF1α during cellular hypoxia by decreasing the stability of the protein, thus achieving the purpose of inhibiting tumor growth. In HCC, the down-regulated HACE1 was closely related to poor survival outcomes, while overexpressed or demethylated HACE1 inhibited proliferation and migration ability of HCC cells [30, 38]. All above indicated that HACE1 might be a potential therapeutic target in HCC patients. C-type lectin domain family 1 member B (CLEC1B), a tumor platelet-related molecule secreted by activated platelets in the peri-tumor area, could affect thrombus formation and hematogenous metastasis of tumors through interactions with podoplanin [39]. Blocking the interaction might be a promising therapeutic strategy for preventing pulmonary metastases from osteosarcoma [40]. Cordycepin treatment effectively reduced the proliferation and migration ability of gastric cancer cells by upregulating CLEC1B [41]. Recently, CLEC1B was confirmed to be remarkably downregulated and related to tumor hemorrhage in HCC, indicating that CLEC1B could be served as a potential target for PD-L1/PD1 immunotherapy [28, 42]. Therefore, to further determine the antitumor effect of CLEC1B, we explored the expression level of CLEC1B in HCC cell lines and a generalized low expression was found. Subsequently, we overexpressed CLEC1B in HuH7 cells and observed a significant inhibition of cell proliferation and migration ability. All these suggested that CLEC1B might be a promising molecule for antitumor immunotherapy in HCC patients. As for COLEC12, it was increased in osteosarcoma and remarkably associated with poor survival outcomes [43], while there are few relevant studies in HCC and further research is needed.

In previous studies, a large number of signatures have been identified for predicting survival outcomes in HCC patients [44–49]. Compared with these signatures, the novel three-gene signature in our current study has some new features: first, the signature constructed based on TDT and TIME is more accurate for HCC monitoring and treatment in clinical practice; second, the signature is validated by qRT-PCR analysis in a small size clinical cohort, ensuring its clinical relevance. Finally, the three-gene signature contains fewer genes and is easier to implement clinically. Undeniably, our current study has some restrictions. The great diversity of HCC and the mechanism of recurrence after treatment may reduce the performance of the signature. Moreover, the small sample size limits the validation of the model, and future multicenter randomized controlled studies are required to evaluate this signature. In addition, the specific mechanisms of the three genes in HCC, especially for CLEC1B, are still not well understood and more in vivo and in vitro experiments are needed in the future.

In summary, a prognostic three-gene signature based on TDTRGs and IRGs was constructed in our current study, which could effectively help clinicians classify HCC patients for prognosis prediction and individualized immunotherapies.

## Supplementary Information

**Additional file 1: Figure S1**. Clinical characteristics evaluation by the signature. Violin plots showing that higher risk scores were linked to later grade (A), T stage (B), advanced TNM stage (C) and recurrence (D).

**Additional file 2: Figure S2**. Prognostic significance of this three-gene signature in TCGA. Kaplan-Meier plot for HCC patients with different (A) age, (B) Gender, (C) Grade, (D)T stage, (E) Cirrhosis, (F) TNM stage, (G) M stage, (H) N stage, (I) vascular invasion status, (J) recurrence status, and (K) AFP value.

**Additional file 3: Table S1**. The sequences of the qPCR primers used in this study. **Table S2**. Clinical characteristics of HCC patients involved in TCGA, ICGC, and the clinical cohort.

## Data Availability

All data generated or analyzed during the present study was downloaded from TCGA database, ICGC database, GEO database, and HPA database. The original CT values of qRT-PCR in the clinical cohort are shown in the Original Data. We can find the datasets analyzed in this study in the https://xena.ucsc.edu and https://dcc.icgc.org/projects/LIRI-JP.
