# Supplementary figures and images for: A novel tumor doubling time-related immune gene signature for prognosis prediction in hepatocellular carcinoma
Source: Cancer Cell Int. 2021 Oct 9;21:522. doi: 10.1186/s12935-021-02227-w (PMC8502295; doi:10.1186/s12935-021-02227-w)

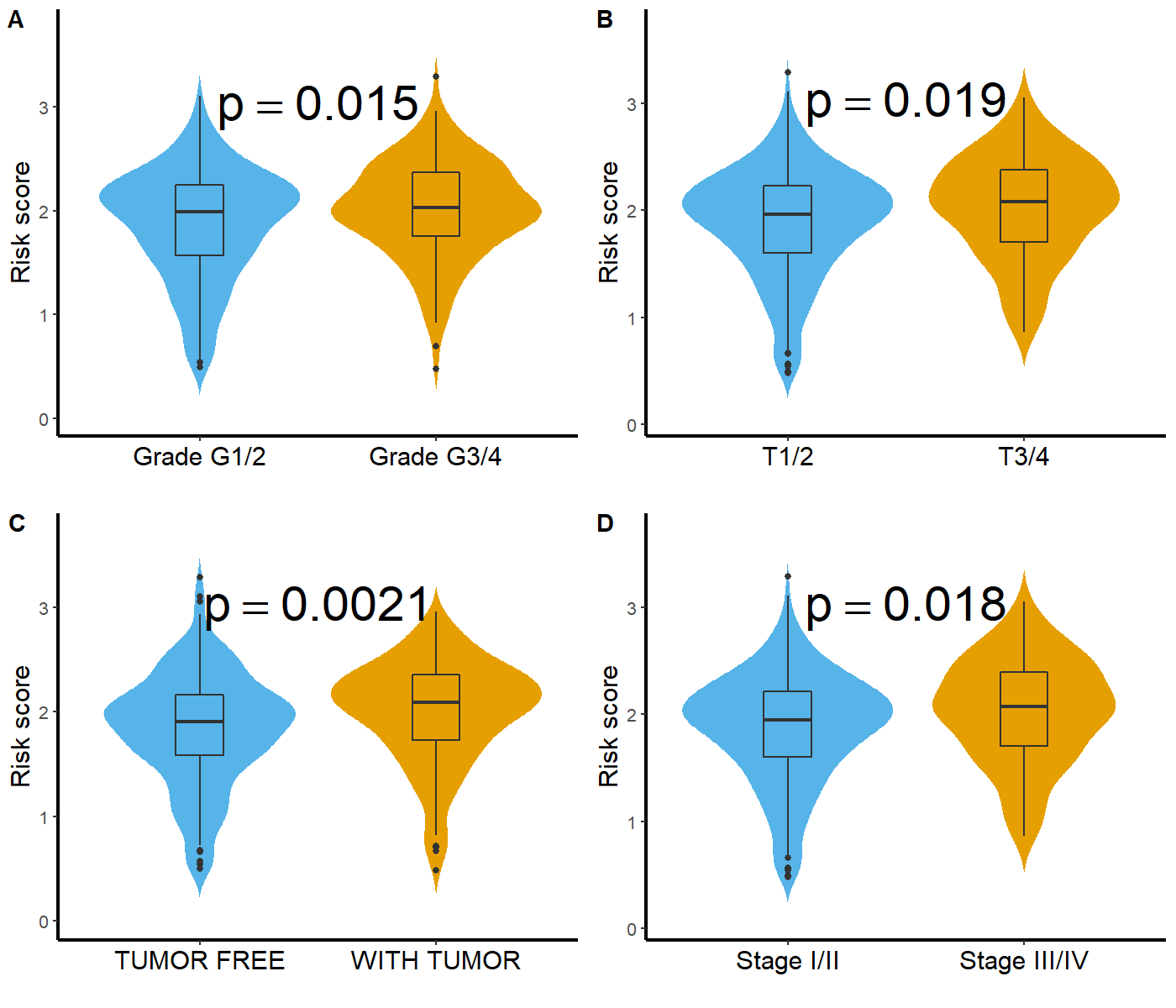

Supplement: Supplementary file 1 — Additional file 1: Figure S1. Clinical characteristics evaluation by the signature. Violin plots showing that higher risk scores were linked to later grade (A), T stage (B), advanced TNM stage (C) and recurrence (D). [file 12935_2021_2227_MOESM1_ESM.png]

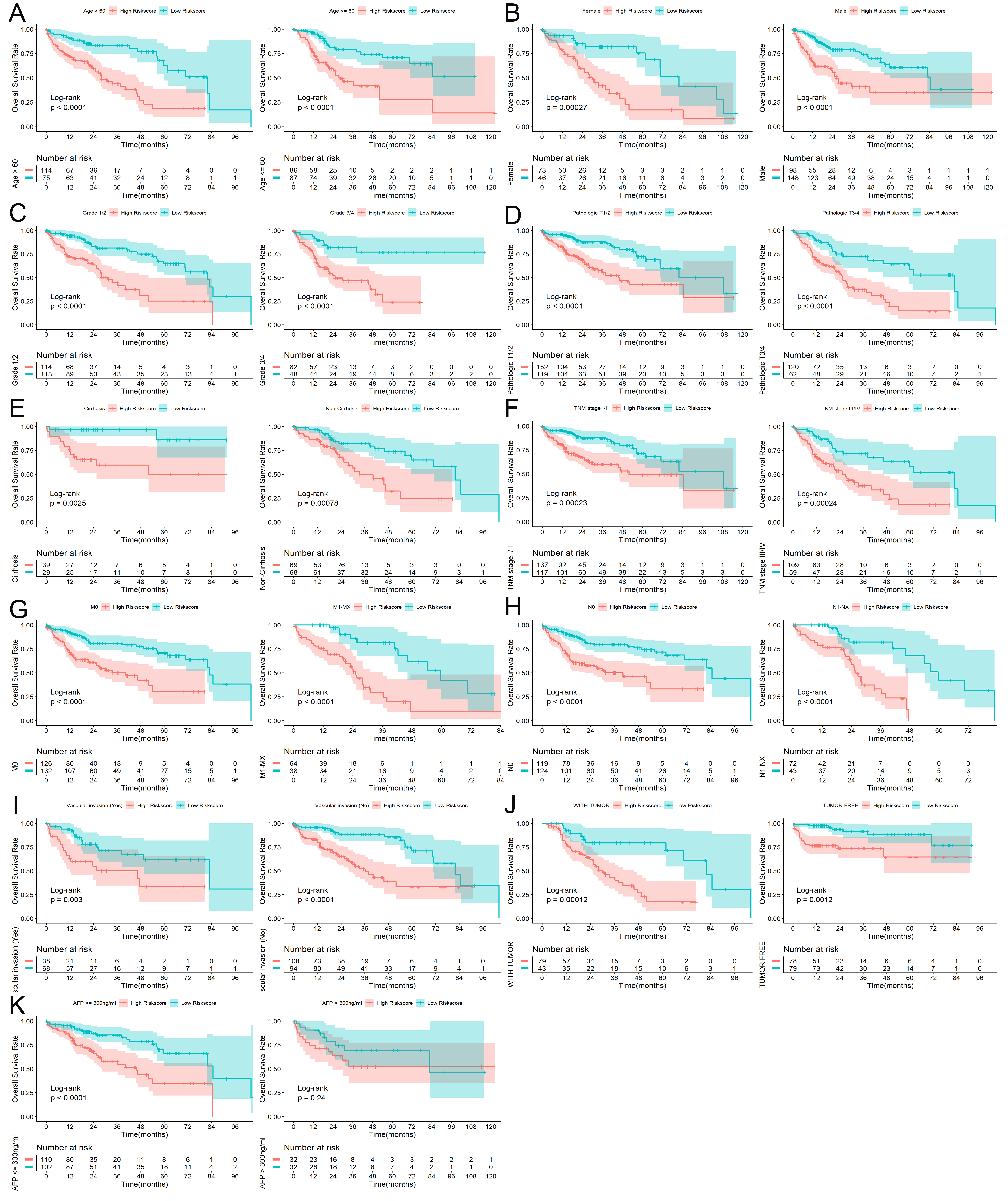

Supplement: Supplementary file 2 — Additional file 2: Figure S2. Prognostic significance of this three-gene signature in TCGA. Kaplan-Meier plot for HCC patients with different (A) age, (B) Gender, (C) Grade, (D)T stage, (E) Cirrhosis, (F) TNM stage, (G) M stage, (H) N stage, (I) vascular invasion status, (J) recurrence status, and (K) AFP value. [file 12935_2021_2227_MOESM2_ESM.tif]
