# Supplementary material for: A novel tumor doubling time-related immune gene signature for prognosis prediction in hepatocellular carcinoma
Source: Cancer Cell Int. 2021 Oct 9;21:522. doi: 10.1186/s12935-021-02227-w (PMC8502295; doi:10.1186/s12935-021-02227-w)
Supplement: Supplementary file 3 — Additional file 3: Table S1. The sequences of the qPCR primers used in this study. Table S2. Clinical characteristics of HCC patients involved in TCGA, ICGC, and the clinical cohort. [file 12935_2021_2227_MOESM3_ESM.docx]

Table. S1 The sequences of the qPCR primers used in this study

| Gene | Forward primer | Reverse primer |
| --- | --- | --- |
| HACE1 | AGTTGCCCGAGGATAATGAAAC | TCCACCGATCCACAATTTGCT |
| CLEC1B | AGCGCAATTACCTACAAGGTG | CTTCCCATGTTAAGTTGTGCCT |
| COLEC12 | AATCCTTCGGTTACAAGCGGT | ACTGTGATTGTTAGCAAGGCAC |
| β-ACTIN | CGTGGGCCGCCCTAGGCACCA | TTGGCTTAGGGTTCAGGGGGG |

Table S2 Clinical characteristics of HCC patients involved in TCGA, ICGC and the clinical cohort.

|  | TCGA cohort  (N=365) | ICGC cohort  (N=227) | Independent cohort  (N=59) |
| --- | --- | --- | --- |
| Gender Male | 119 | 61 | 45 |
| Female | 246 | 166 | 14 |
| Age ≤60 years | 173 | 49 | 27 |
| >60 years | 192 | 178 | 32 |
| Grade G1/2 | 230 |  | 35 |
| G3/4 | 130 |  | 24 |
| unknown | 5 |  |  |
| TNM Stage I/II | 254 | 140 | 42 |
| III/IV | 87 | 87 | 17 |
| unknown | 24 | 0 | 0 |
| Vascular Invasion Yes | 106 |  |  |
| No | 205 |  |  |
| unknown | 5 |  |  |
| Recurrence With tumor | 122 |  | 29 |
| Tumor free | 161 |  | 30 |
| unknown | 82 |  | 0 |
| Cirrhosis With | 68 |  | 6 |
| Without | 141 |  | 53 |
| unknown | 156 |  | 0 |
| HBV or HCV Infection |  |  |  |
| Yes | 149 |  | 41 |
| No | 203 |  | 18 |
| unknown | 13 |  | 0 |
| ****Child-Pugh A**** | 216 |  | 57 |
| B | 21 |  | 2 |
| C | 1 |  | 0 |
| unknown | 127 |  | 0 |
